# The effect of DMSO on *Saccharomyces cerevisiae* yeast with different energy metabolism and antioxidant status

**DOI:** 10.1038/s41598-024-72400-4

**Published:** 2024-09-20

**Authors:** Agata Święciło, Ewa Januś, Anna Krzepiłko, Monika Skowrońska

**Affiliations:** 1 Department of Environmental Microbiology, University of Life Sciences in Lublin, Leszczyńskiego 7, 20-069 Lublin, Poland; 2 Department of Cattle Breeding and Genetic Resources Conservation, University of Life Sciences in Lublin, Akademicka 13, 20-950 Lublin, Poland; 3 Department of Biotechnology, Microbiology and Human Nutrition, University of Life Sciences in Lublin, Skromna 8, 20-704 Lublin, Poland; 4 Department of Agricultural and Environmental Chemistry, University of Life Sciences in Lublin, Akademicka 15, 20-950 Lublin, Poland

**Keywords:** DMSO, *Saccharomyces cerevisiae*, Xenobiotic, Spot test, ESR, Oxidative stress, Biochemistry, Cell biology, Microbiology

## Abstract

We studied the effect of dimethyl sulfoxide (DMSO) on the biochemical and physiological parameters of *S. cerevisiae* yeast cells with varied energy metabolism and antioxidant status. The wild-type cells of varied genetic backgrounds and their isogenic mutants with impaired antioxidant defences (Δ*sod* mutants) or response to environmental stress (ESR) (Δ*msn2*, Δ*msn4* and double Δ*msn2msn4* mutants) were used. Short-term exposure to DMSO even at a wide range of concentrations (2–20%) had little effect on the metabolic activity of the yeast cells and the stability of their cell membranes, but induced free radicals production and clearly altered their proliferative activity. Cells of the Δ*sod1* mutant showed greater sensitivity to DMSO in these conditions. DMSO at concentrations from 4 to 10–14% (depending on the strain and genetic background) activated the ESR programme. The effects of long-term exposure to DMSO were mainly depended on the type of energy metabolism and antioxidant system efficiency. Yeast cells with reduced antioxidant system efficiency and/or aerobic respiration were more susceptible to the toxic effects of DMSO than cells with a wild-type phenotype and respiro-fermentative or fully fermentative metabolism. These studies suggest a key role of stress response programs in both the processes of cell adaptation to small doses of this xenobiotic and the processes related to its toxicity resulting from large doses or chronic exposure to DMSO.

## Introduction

Dimethyl sulfoxide (Me_2_SO; commonly referred to as DMSO) is a natural compound present in low nanomolar concentrations in a variety of environments, mainly in water, but also in the atmosphere or in sediments^1–4^. It is formed abiotically from photooxidation of dimethyl sulfide (DMS) and in the metabolic pathways of bacteria and algae in aquatic ecosystems. It is believed to be one of the intermediates of the biogeochemical pathway of sulfur^5^. DMSO is produced anthropogenically in large amounts (annual production of DMSO is about 50,000 metric tons) [https://www.transparencymarketresearch.com/dimethyl-sulfoxide-market.html] and used on a large scale in various branches of industry, such as the pharmaceutical, electronic, chemical, and agricultural industries. As a by-product or residue following technological processes, DMSO can in some cases make up as much as 20 wt% of industrial wastewater^6^. While technologies have been developed to effectively remove DMSO from industrial wastewater, below a certain level of DMSO (usually 0.24% v/v) they are unprofitable, and therefore DMSO residues usually enter the sewage system and environment, raising the level of environmental DMSO.

As a polar aprotic solvent which dissolves both polar and apolar compounds, with an exceptional ability to penetrate biological membranes and cellular barriers, DMSO is often exploited in research in the fields of biochemistry and cell biology^7^. These characteristics of DMSO also enable its use in medical diagnostics, for evaluating changes in the barrier properties of human and to facilitate the introduction of therapeutic drugs into the body in the treatment of various conditions^8,9^. As a solvent of pharmaceutical compounds, it is usually used at concentrations of 0.1–5% (v/v)^10^. More recent studies have shown that as an ingredient of pharmaceutical preparations, DMSO can also affect their pharmacological activity. As an adjuvant inhibiting end-cell differentiation in tumours, it can effectively enhance anticancer therapy^11^. Moreover, DMSO itself has various therapeutic and pharmaceutical properties, such as anti-inflammatory, radioprotective, and local and systemic analgesic, antibacterial, antifungal, antiviral and anticancer properties^12,13^.

In scientific research and in cryosurgery, DMSO is used as a cryoprotectant for storing various types of cells, tissues, and cell- and tissue-based products for long periods of time at low temperatures. For this purpose DMSO is usually used at concentrations from 5 to 10%, and occasionally even 15% (v/v)^14^.

It has been suggested that what underlies the phenomenon of cryoprotection and the anti-inflammatory properties of DMSO is its ability to scavenge free radicals, resulting from its specific ability to react with hydroxyl radicals (OH·). DMSO usually exhibits these properties at low concentrations ≤ 0.1%; however, the effect is strongly dependent on the cellular research model^15,16^.

Apart from the examples presented above of the protective effects of DMSO on organisms and cellular systems, the literature also contains examples of its neutral or even toxic effects. The harmful effects of DMSO were previously thought to be associated with high concentrations of the compound (usually > 10%)^17^ and mainly involved damage to the cell membrane via plasma membrane pore formation, but there have been increasing reports of its harmful effects even at low concentrations. DMSO applied at a concentration of 1% v/v for 48 h is toxic for human lens epithelial cells, effectively reducing their viability by promoting apoptosis and upregulating the apoptotic promoter Bax in these cells^18^. Similarly, treatment of H9c2 cardiomyoblasts and MCF-7 breast cancer cells with DMSO at a concentration of 3.7% leads to induction of apoptosis, associated with mitochondrial dysfunction, oxidative stress, and necrosis, thus reducing the survival rate of these cells^19^. At low concentrations (2–4%), DMSO induces retinal apoptosis in both in vivo and in vitro studies^20^. It also exhibits toxic activity at concentrations above 3% at exposure times longer than 24 h in planarians *Dugesia japonica*^21^, at 2.5–7.5% against various strains of the dermatophyte *Trichoderma mentagrophytes* and the yeast *Candida albicans*^22^, and at 0.5–7% against the plant pathogen *Botrytis cinerea*^23^.

Given the widespread use of DMSO in various areas of human life, owing to its beneficial physical, chemical and biological properties, and in view of the inconsistent and often conflicting reports on its safety for organisms and the environment, information about its nonspecific, potential harmful cellular effects is extremely important. These issues were investigated in this work using the yeast *Saccharomyces cerevisiae*.

*S. cerevisiae* yeast is a recognized model organism. The species is one of the best known in terms of the genetics, biochemistry and physiology of eukaryotic organisms. In this study, we used wild-type strains and their isogenic mutants with modified responses to harmful environmental factors. These were cells of ∆*sod1* and ∆*sod2* strains without activity of superoxide dismutases – enzymes which scavenge superoxide anion radical from the cytoplasm and mitochondrial matrix, respectively, as well as strains lacking activity of Msn2p and Msn4p, which activate the environmental stress response programme. Strains with reduced antioxidant system efficiency (∆*sod1* and ∆*sod2* mutants) were used due to their hypersensitivity to conditions inducing oxidative stress, which often accompanies symptoms of cytotoxicity of xenobiotics^24–27^. By manipulating culture conditions, such as oxygen availability and/or carbon sources (as the sole carbon and energy source), a population of cells with fully respiratory or fermentative metabolism or even a mixed respiratory-fermentative metabolism was obtained. In addition, by using ethidium bromide to mutagenize respiratory competent cells, i.e. those capable of aerobic respiration, we obtained mutants without functional mitochondria, i.e. with permanent fermentative metabolism. Rearrangement of energy metabolism, induced by culture conditions or by the effect of a specific mutagen, entails major genetic, biochemical and physiological changes which can affect the overall sensitivity of yeast cells to environmental factors. A phenotypic characterization of the strains used in the study is given in Supplementary Table S1.

The aim of the study was to determine the effects of short-term and long-term exposure to DMSO at high concentrations characteristic of the anthropogenic environment (2–20% v/v/) on *S. cerevisiae* yeast. We studied the effect of one-hour exposure to DMSO on the biochemical and physiological parameters of yeast cells, such as metabolic activity, the functionality of their cytoplasmic membranes, and their ability to proliferate, as well as whether this xenobiotic is able to modulate the genetic/metabolic activity of the cell (i.e. to activate the environmental stress response (ESR) programme and induce oxidative stress). In the second part of the study, we compared the effects of long-term (four-day) exposure to DMSO on yeast cells with varying antioxidant system efficiency (wild type cells and *sod1* and *sod2* mutants) and with varying energy metabolism (aerobic, mixed respiratory-fermentative and fully fermentative metabolism) to identify cellular targets which are particularly sensitive to this xenobiotic.

## Results

### The short-term effect of DMSO on *S. cerevisiae* yeast cell

The test of short-term (1 h) effects involved determination of the reducing power of the yeast cells as an indicator of their metabolic activity, catalase T activity as an indicator of general stress response, electrical conductivity as a marker of cell membrane damage, and survival rate as a marker of their proliferative capacity measured as the ability of cells to form colonies on solid media. We also determined the level of superoxide anion radicals generated by DMSO exposure.

**Fig. 1:**
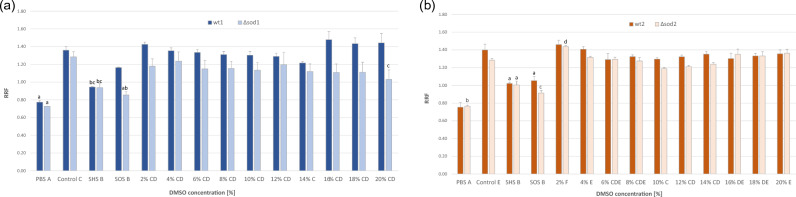
Metabolic activity of yeast cells in the presence of DMSO (**a**) for cells of wt1 and Δ*sod1* strains; (**b**) for cells of wt2 and Δ*sod2* strains.  Explanations: wt1 – wild-type strain SP4, Δ*sod1* – its isogenic mutant lacking cytoplasmic dismutase activity, wt2 – wild-type strain EG 103, Δ*sod2* – mutant isogenic to wt2 lacking mitochondrial superoxide dismutase activity, PBS – phosphate-buffered saline, Control – cells not exposed to DMSO and any other stress factor, SHS – cells subjected to strong heat stress (48 °C for 30 min), SOS – cells subjected to strong oxidative stress (0.75% H_2_O_2_ for one hour), RRF – resazurin reduction factor. Data are presented as the mean ± SEM of three biological experiments with four technical repeats (n = 12). Capital letters (A, B, C, D, E, F) denote the means that form homogenous groups (HSD–Tukey post–hoc test for multivariate ANOVA) within treatment (PBS, HS, OS, DMSO) regardless of the yeast genotypes. Lowercase (a, b) denote significant difference within-strains, vs. control (0%) DMSO.

The reducing power of yeast of two wild-type strains (wt1 and wt2) and mutants lacking superoxide dismutase activity in the cytosol (Δ*sod1*) and in the mitochondrial matrix (Δ*sod2*) under the influence of DMSO was tested using the resazurin assay. DMSO was applied in a wide range of concentrations (from 2 to 20% (v/v)). Samples in which yeast were incubated with strong stress factors – heat and oxidative stress – were used as a positive control. The results are shown in Fig. 1.

The RRF value was sharply reduced in cells of all strains following the application of severe stress: heat stress (48 °C for 30 min) or oxidative stress (induced by a 0.75% hydrogen peroxide). However, in the case of cells of the wt1 strain subjected to oxidative stress the change was not statistically significant. DMSO applied in a wide range of concentrations from 2 to 20% (v/v) did not reduce the RRF value for cells of the wild-type strains: wt1*,* wt2 or the Δ*sod2* mutant. The RRF for the latter was even significantly higher than for the control cells at concentration of 2% DMSO.

DMSO also had no negative effect on the reducing power of cells of the Δ*sod1* mutant, except at the highest concentration, at which the RRF value was about 20% lower than in the control.

**Fig. 2:**
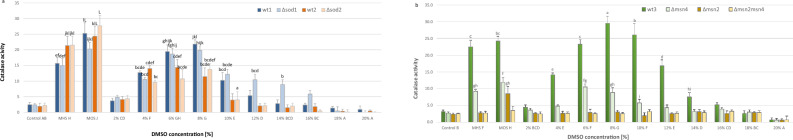
Catalase activity of yeast cells in the presence of DMSO (**a**) for cells of wt1*,* wt2, Δ*sod1*, Δ*sod2* strains; (**b**) for cells of wt3, Δ*msn4*, Δ*msn2* and Δ*msn2msn4* strains.  Explanations: Control, wt1, wt2, Δ*sod1*, Δ*sod2*, as in Fig.  1, wt3—wild-type strain BY4741; Δ*msn2*, Δ*msn4*, Δ msn2msn4 —its isogenic mutants lacking Msn2p, Msn4p or both transcription factors activating the general stress response, MHS – cells subjected to mild heat stress (37 °C for 30 min), MOS – cells subjected to mild osmotic stress (0,3 M NaCL for one hour), Data are presented as the mean ± SEM of three biological experiments with four technical repeats (n = 12). Capital letters (A, B, C, D, E, F) denote the means that form homogenous groups (HSD–Tukey post–hoc test for multivariate ANOVA) within treatment (Control, MHS, MOS, DMSO) regardless of the yeast genotypes. Lowercase (a, b, c, d, e) denote significant difference within-strains, vs. control (0%) DMSO.

Next, the ability of DMSO to induce the ESR programme was tested in yeast cells of wild-type strains, sod mutants, and stress response-defective mutants. The activity of cytoplasmic catalase T was used as a marker of the stress response^28^. In this case, mild heat shock (MHS) and osmotic shock (MOS) served as positive controls (Fig. 2).

In conditions of mild heat shock or osmotic shock, catalase activity increased in the cells of the wild-type strains and the Δ*sod* mutants relative to the untreated control, from six-fold to 12-fold, which indicates activation of the ESR programme in these cells. DMSO induced a similar response in the wt2 and Δ*sod2* strains at concentrations of 4–8%, and in wild-type strains wt1 and wt3 at concentrations in the range of 4–10%. In the cells of the Δ*sod1* mutant, there was a significant increase in catalase activity relative to the control for a wider range of DMSO concentrations – 4–14%. In the case of cells lacking the activity of transcription factor Msn4p, a smaller increase – about 3.5-fold – in the activity of the enzyme was noted in samples incubated in conditions of mild heat stress or mild osmotic stress and in the presence of DMSO at concentrations of 6–10%. In the cells of the remaining ESR-defective mutants (Δ*msn4* and Δ*msn2msn4*), no changes in catalase activity were induced either by mild environmental shock or by the presence of DMSO.

**Fig. 3:**
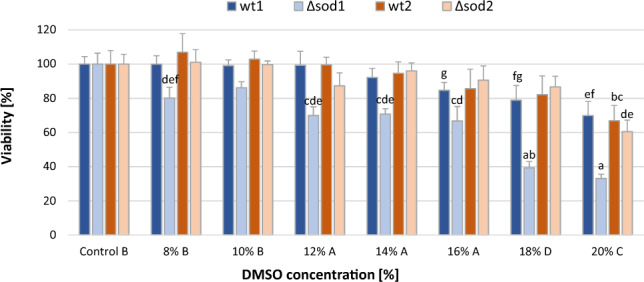
Effect of DMSO on the viability of yeast cells.  Explanations: Control, wt1, wt2, Δ*sod1*, Δ*sod2*, as in Fig. 1. Data are presented as the mean ± SEM of three biological experiments with four technical repeats (n = 12). Capital letters (A, B, C, D) denote the means that form homogenous groups (HSD–Tukey post–hoc test for multivariate ANOVA) within DMSO treatments regardless of the yeast genotypes. Lowercase (a, b, c, d) denote significant difference within-strains: a, wt1 DMSO treatment, vs. control; b, Δ* sod1* strain DMSO treatment, vs. control; c, wt2 DMSO treatment, vs. control DMSO; d, Δ *sod2* DMSO treatment, vs. control.

In the next stage of the study, the survival of yeast in the presence of DMSO was determined based on their ability to form colonies on a solid medium (Fig. 3). The yeast showed relatively high resistance to one-hour exposure to DMSO. Only incubation with relatively high concentrations of this solvent led to a reduction in the survival of these cells. Cells without cytoplasmic superoxide dismutase activity – Δ*sod1s* – proved to be the most sensitive to DMSO. At a concentration of 8% v/v, DMSO reduced their survival rate by about 20%. Lower concentrations of DMSO did not affect their ability to form colonies (data not shown). Increasing the concentration of the solvent to 16% (v/v) reduced the survival rate of these cells by about 33%, while 20% (v/v) DMSO reduced their viability by about 70%. The remaining strains were less susceptible to the antiproliferative effect of DMSO. In the case of the wild-type strain wt1, this effect was only manifested in the presence of DMSO at a concentration of 16% or higher. The other two strains, i.e. the wild-type strain wt2 and its isogenic mutant Δ*sod2*, did not respond with a significantly decreased survival rate until the DMSO concentration reached 20%. In this case, their viability was reduced on average by 36.3% relative to the untreated control cells.

**Fig. 4:**
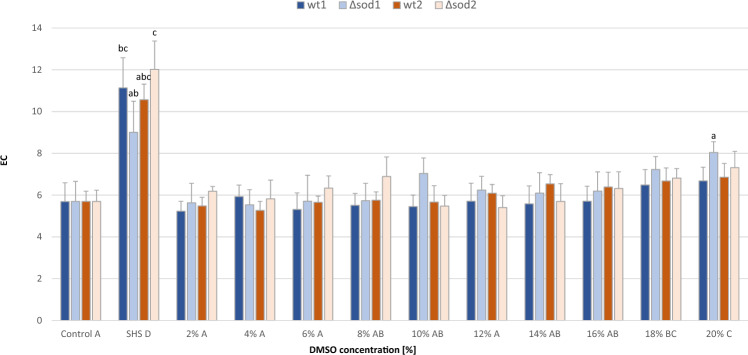
Effect of DMSO on the permeability of cytoplasmic membranes.  Explanations: Control, SHS, wt1, wt2, Δ*sod1*, Δ*sod2*, as in Fig. 1. Data are presented as the mean ± SEM of three biological experiments with four technical repeats (n = 12). Capital letters (A, B, C, D) denote the means that form homogenous groups (HSD–Tukey post–hoc test for multivariate ANOVA) within treatment (HS, DMSO) regardless of the yeast genotypes. Lowercase (a, b, c, d, e) denote significant difference within-strains, vs. control (0%) DMSO.

To investigate the causes of the cytotoxicity observed, the integrity of cytoplasmic membranes was examined, as they are postuladed target of the harmful effects of DMSO^17^. For this purpose the electrical conductivity (EC) of a solution in which yeast cells were suspended was measured (Fig. 4). An increase in the EC value indicates an increase in the concentration of electrolytes originating in the yeast cells. As the effect of DMSO was tested in a wide range of concentrations from 2 to 20%, first the EC value was determined for aqueous solutions of this reagent alone. They ranged from 1.04 to 1.20 µS/m (the value obtained for deionized water was 0.96 µS/m), so it was concluded that the presence of DMSO has a marginal effect in this assay. Heat stress caused an approximately twofold increase in EC for the cells of all strains analysed. Among these, cells of the Δ*sod1* mutant had the lowest EC value (1.4 times that of the control). The presence of DMSO even at a relatively high concentration (up to 20%) had no significant effect on the EC of the environment of the cells of the wt1, wt2 and Δ*sod2* strains. In the case of the Δ*sod1* strain, only the highest concentration of DMSO caused a significant increase in electrical conductivity above the value for the control, but it was about 10% lower than the value for cells subjected to heat stress. Thus, it can be concluded that short-term (one-hour) incubation of the cells of the wild-type yeast strains and Δ*sod2* strain with DMSO does not significantly affect cell membrane permeability. In the case of the Δ*sod1* yeast strain, only the highest concentration of DMSO (20%) negatively affects membrane functions.

**Fig. 5:**
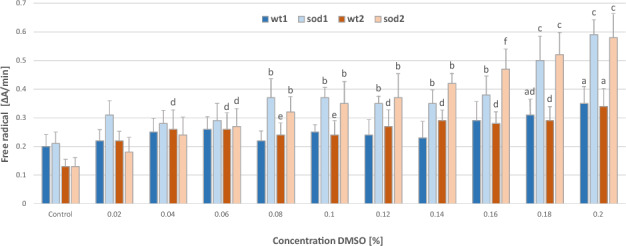
Superoxide radical anions level in yeast cells exposed to DMSO.  Explanations: Control, wt1, wt2, Δ*sod1*, Δ*sod2*, as in Fig.  1. Data are presented as the mean ± SEM of three biological experiments with three repeats (n = 9). Lowercase (a, b, c, d, e, f) denote significant difference within-strains, vs. control (0%) DMSO.

Greater sensitivity of dismutase-deficient strains to the harmful effects of DMSO suggests that DMSO at the subcellular level is capable of inducing free radicals, including superoxide anion. The level of superoxide anions in yeast cells was determined using the NBT reduction assay (Fig. 5). The level of superoxide anion radicals increased in proportion to the DMSO concentration, but in *sod* mutant cells this effect was much stronger.

### Long-term effect of DMSO on *S. cerevisiae* yeast

**Fig. 6:**
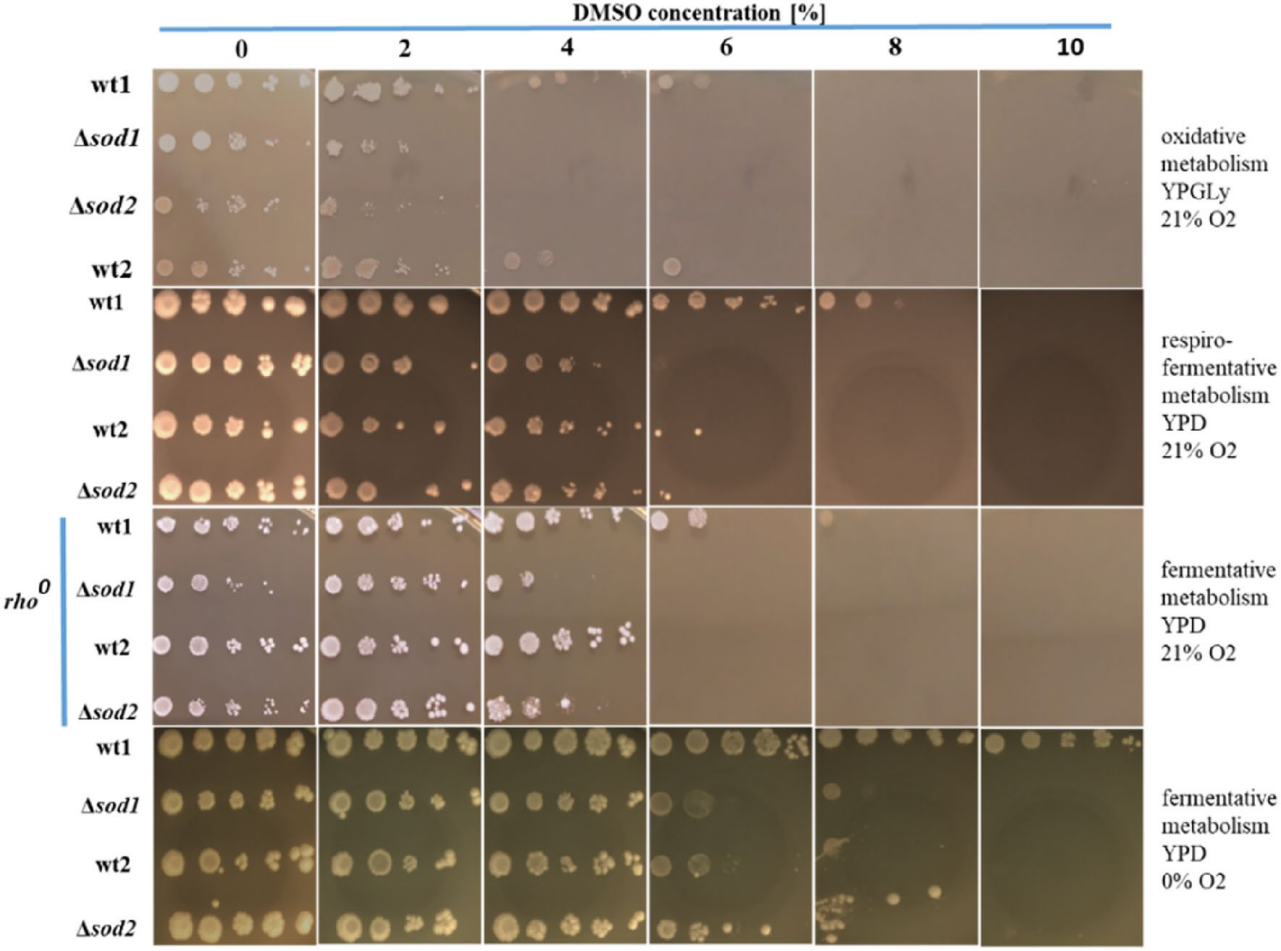
Sensitivity of *S. cerevisiae* yeast cells with varying energy and antioxidant status to DMSO.  Explanations: wt1, wt2, Δ*sod1*, Δ*sod2*, as in Fig.  1. *rho°* – respiratory deficient mutants, lacking functional mitochondria, YPGly – medium containing glycerol as the sole energy and carbon source, YPD – medium containing glucose as the sole energy and carbon source. Representative results from a set of three experiments are shown. Ten-fold dilutions: from left to right (10^0^–10^–4^).

The level of resistance of yeast cells to DMSO in relation to their energy metabolism and the efficiency of their antioxidant system was analysed. The long-term (4-day) effects of DMSO on yeast were tested by spot-plating suspensions of yeast cells on solid media containing various concentrations of DMSO (Fig. 6).

Long-term exposure of *S. cerevisiae* yeast to DMSO at concentrations above 4% leads to a reduction or complete inhibition of their growth (depending on the genetic background of the strain and their energy metabolism). Reduced growth is manifested in this case as growth in the form of spots only when dense cell suspensions have been plated or spots of smaller diameter than in the control.

Cells with aerobic respiration growing on a medium with glycerol (YPGly) are more sensitive to DMSO than those obtaining energy through a mixed respiro-fermentative (growing on YPD medium with standard aeration) or fully fermentative metabolic pathway. DMSO at a concentration of just 2% severely limits their growth, and at 4% completely inhibits the growth of the Δ*sod1* and the Δ*sod2* mutant strains and substantially reduces the growth of the wt1 and wt2 wild-type strains. Yeast with respiro-fermentative metabolism (second row of photos in Fig. 6) have a similar level of resistance to DMSO as the cells of respiratory-deficient strains (*rho*^*0*^ mutants) (third row of photos). These yeasts grow on media containing up to 4% (v/v) DMSO. Mutants lacking cytoplasmic superoxide dismutase activity (*Δsod1*) and mitochondrial superoxide dismutase activity (*Δsod2*) show poorer growth than the isogenic wild-type strains, with the *Δsod1* mutant appearing to be more sensitive to the harmful effects of DMSO than the *Δsod2* mutant. A similar pattern of sensitivity on DMSO of *rho*^+^ and *rho*^*0*^ cells generating energy partially or completely through fermentation under aerobic conditions (second and third row of photos in Fig. 6) suggests that mitochondrial functions do not play a major role in the mechanisms of toxicity of this xenobiotic. Yeast cells with the fermentative metabolism, induced by the lack of oxygen showed a higher level of resistance to DMSO than yeast growing in the presence of oxygen (regardless of their type of energy metabolism).

**Fig. 7:**
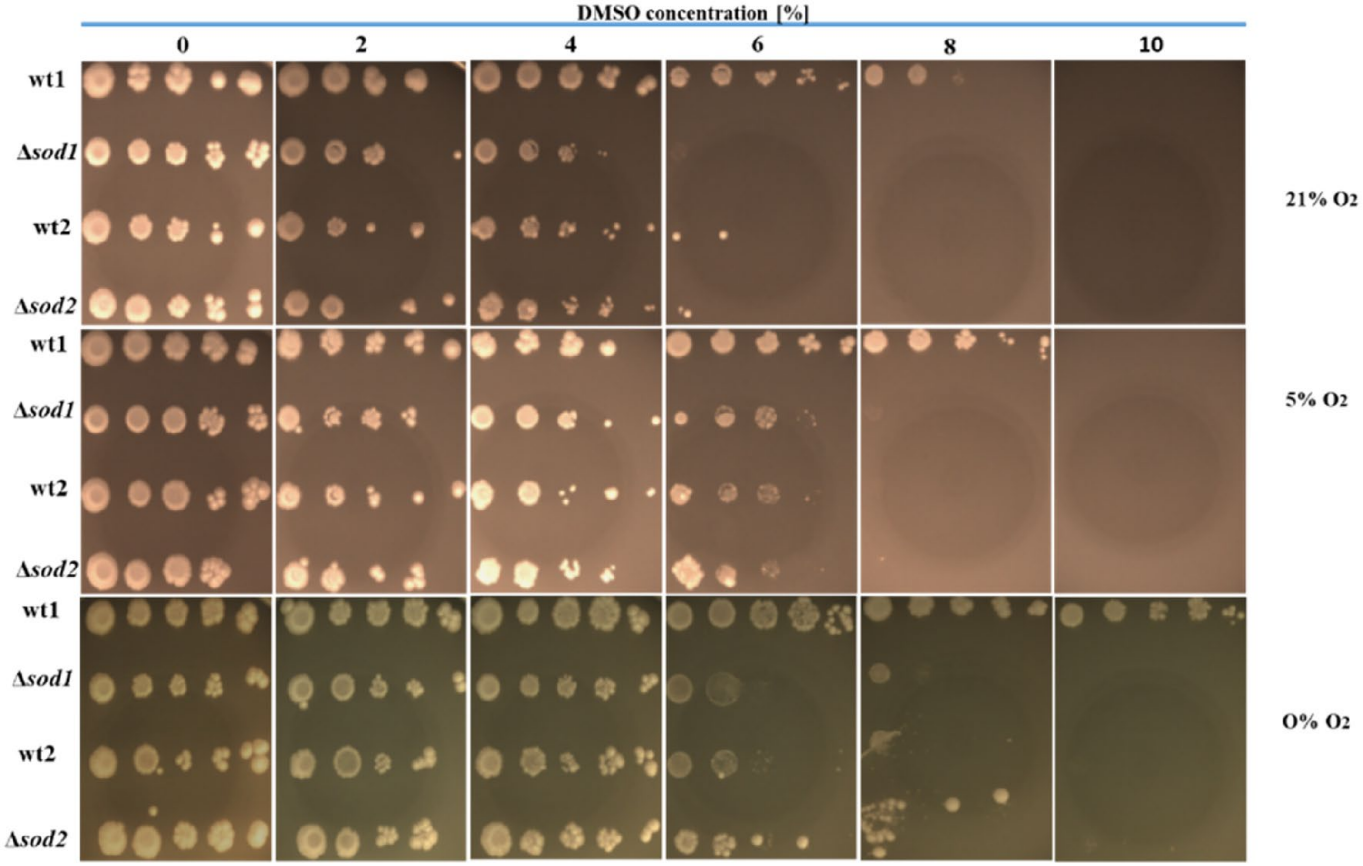
Sensitivity of *S. cerevisiae* yeast cells with varying antioxidant status to DMSO at condition of normoxia, hypoxia and anoxia.  Explanations: wt1, wt2, Δ*sod1*, Δ*sod2*, as in Fig.  1. Representative results from a set of three experiments are shown. Ten-fold dilutions: from left to right (10^0^–10^–4^).

As mentioned earlier, the yeast *S. cerevisiae* can live in environments with varied levels of oxygen (from the complete absence of oxygen (anoxia) to the typical concentration for atmospheric air, i.e. 21% (normoxia) to 100% oxygen content (hyperoxia)^29,30^. The availability of oxygen is one of the main factors that determines the scope of use of both ways of generating energy (fermentation and/or respiration). The results of the yeast growth test obtained for normoxia (YPD, 21% O_2_) and anoxia conditions (YPD, 0% O_2_) (shown in Fig. 6) are compared with the test results for hypoxia conditions (YPD, 5% O_2_) (Fig. 7).

Reducing the oxygen concentration in the environment from normoxia (21% O_2_) to hypoxia (5% O_2_) restores growth to dismutase-deficient mutants and the cells of wild-type strain wt2 in a medium containing 6% DMSO and improves the growth of cells of the wild-type strain at 8% DMSO (Fig. 7). Conditions of anoxia (a total lack of oxygen) completely abolish the sensitivity of cells of wild-type strain wt1 to DMSO in a 8–10% range of concentrations and improves the growth of cells of the remaining strains in media containing 6–8% DMSO.

In the next experiment, the effect of a low–molecular-weight antioxidant (ascorbate) has been investigated on yeast cells grew in DMSO presence. As can be seen in the Supplementary Fig. S1, supplementation of media with this compound did not affect the growth parameters of either dismutase-deficient mutants or wild-type strain wt2, but slightly improved the growth of wild-type strain wt1 growing in the presence of 8% DMSO. Improvement in the growth of cells of wild-type strain wt1 in the presence of ascorbate was noted only at the lowest concentration (10 mM).

## Discussion

The data presented in the introduction indicate that DMSO alone can exert various cellular effects, depending on the experimental design, test organism, and concentration. It is generally believed that low concentrations of this solvent are safe and have antioxidant and/or protective effects for cellular structures and processes, whereas high concentrations can be harmful. The research presented in this paper confirms these assumptions and at the same time attempts to explain the reasons for its multifaceted nature.

After one hour of exposure to DMSO, we determined the metabolic activity of the yeast cells, the stability of the cytoplasmic membranes, and the cells’ ability to proliferate, expressed as survival rate (i.e. the ability to form colonies on solid medium). There was almost no change in the first two parameters in the presence of a wide range of concentrations of DMSO (2–20% v/v DMSO) (Figs. 1, 4). Only cells of the strain without cytoplasmic superoxide dismutase (Δ*sod1*) activity, at the highest concentration of DMSO (20% v/v), had reduced metabolic activity (measured as RRF) and increased permeability of cytoplasmic membranes (determined as EC). However, the values of these parameters differed from the RRF and EC values obtained for cells exposed to severe environmental shock (heat or oxidative), which our previous research has shown to have destructive effects on cell structures, leading to the death of yeast cells^31^. This suggests that DMSO in the analysed range of concentrations does not disturb cellular metabolism and has no negative effects on the cytoplasmic membranes of wild-type or Δ*sod2* mutant yeast cells, while it has a moderate effect on Δ*sod1* mutant cells. The sensitivity of Δ*sod1* mutant cells to high DMSO concentrations may be due to the pro-oxidant properties of this compound, which are expressed in this specific cell environment. Cells of this mutant lack activity of cytoplasmic superoxide dismutase, an enzyme which scavenges superoxide anion radical, and thus are more vulnerable to the harmful effects of reactive oxygen species (ROS), generated both in optimal conditions for yeast growth (mainly as a by-product of oxygen metabolism) and in the presence of pro-oxidants^32^. The concentration of oxygen anion radicals in the presence of just 8% DMSO, was shown to be higher in the cells of the Δ*sod1* mutant than in the control cells and wild-type cells in the same conditions (Fig. 5).

Similar results were obtained by the Sadowska-Bartosz et al.^27^, moreover, they noted in these conditions reduced activity of succinate dehydrogenase (an enzyme particularly susceptible to oxidative damage) and an increased level of the oxidized form of glutathione (a cellular component of redox buffer). As the DMSO concentration increased, there was also an increase in reduced glutathione and a progressive increase in membrane anisotropy (an inverse measure of membrane fluidity), explained as a reaction compensating for the pro-oxidant effects of DMSO on components of cytosol and cell membranes^27^.

One-hour exposure to DMSO in a range of concentrations from 4 to 10 to 14% (depending on the strain and genetic background) led to the activation of the ESR programme (Fig. 2). This is a universal phenomenon which allows microorganisms to adapt to variable and often unfavourable environmental conditions. In *S. cerevisiae* yeast, activation of this programme is mediated by transcription factors Msn2p and Msn4p in response to many different types of environmental stress, such as starvation, heat stress, osmotic stress, alcohol stress, or oxidative stress^33^. A lack of Msn2p activity (as in the case of the Δ*msn2* and Δ*msn2msn4* cells) prevented the activation of this programme, whereas a lack of activity of its homologue Msn4p had less pronounced effects (Fig. 2b). Therefore, the roles of the two factors do not fully overlap, which is confirmed by literature data^34^. The biological purpose of ESR is not to protect against a current stimulus (mild stress) but to ‘prepare’ cells for a subsequent strong stimulus (severe stress). So the ability to trigger this program indicates the potentially dangerous nature of DMSO. Activation of this programme results in resistance to the same factor or to another type of strong stress factor, which is referred to as cross-protection^35,36^. This phenomenon may therefore play an important role in cryoprotection or radioprotection induced by DMSO^37,38^ and can also modify the response of yeast to substances dissolved in DMSO.

The absence of effects of short-term exposure to DMSO on metabolic activity and cell membrane permeability in the wild-type strains and Δ*sod2* mutant (and the minor effect on these parameters in the SOD1-deficient yeast mutant) is in contrast with the effect of this compound on the survival of the cells. DMSO at a concentration equal to or greater than 8% (v/v) significantly reduced the survival of ∆*sod1* mutant cells, while a concentration equal to or greater than 16% had this effect in cells of the wild-type strain wt1, and at 20% on the cells of the wt2 and ∆*sod2* strains (Fig. 3). The reduced survival rate of cells following short-term contact with DMSO seems to be a phenomenon resulting from inhibition of the cell cycle rather than from damage to key cellular structures. Numerous studies confirm that DMSO even at low concentrations can completely arrest or slow down the cell cycle of various types of cells^39–41^. This is also supported by our findings and those of Sadowska-Bartosz et al.^27^ on yeast cell membrane integrity in the presence of DMSO. In both cases, despite the use of different research methodologies, no drastic changes were noted in the functionality of these structures even at high concentrations of DMSO. This is rather surprising given that the main mechanism of the harmful effects of DMSO is generally assumed to be the destabilization of cell and organelle membranes^42,43^. However, both research involving numerical simulations (molecular dynamics) and analyses of the response of cell membranes of living fibroblasts to various concentrations of DMSO indicate that the ultimate effect depends primarily on the concentration of these solvent. Three ranges of effective DMSO concentrations are distinguished, which–irrespective of the type of membrane and its composition–lead to characteristic modifications: membrane loosening, pore formation, and bilayer collapse^7,44^. The first of these effects is induced by relatively low concentrations up to 15% v/v. These concentrations of DMSO does not create pores in the lipid bilayer and does not significantly increase permeability (for water, small ions, and large molecules), while at concentrations from 15% do 20% it leads to the formation of hydrophilic, stable pores, which is linked to an increase in permeability for water and small ions (but membranes remain intact in these conditions and retain their selectivity). Only when DMSO is applied at concentrations higher than 20% (25%, 30% and 40%) does it lead to the formation of numerous extended blebs, suggesting a loss in membrane integrity leading to its destruction. Therefore, our results are in agreement with these observations. However, the lack of clear evidence for cell membrane damage caused by the doses of DMSO used in our study does not rule out the possibility that it acts at the subcellular level.

Next, we tested the effects of long-term exposure to DMSO on yeast cells with varying antioxidant status and energy metabolism. By manipulating culture conditions (as presented in Supplementary Table S2) we obtained populations of yeast cells with fully aerobic metabolism, respiro-fermentative metabolism, and fully fermentative metabolism. In addition, for the purposes of this study we produced respiratory-deficient mutants, which due to damage to mitochondrial functions had obligatory fermentative metabolism. Comparison of the sensitivity of yeasts with different energy metabolism to the toxic effects of DMSO reveals that yeast cells with fully aerobic metabolism were the most sensitive to this xenobiotic, followed by respiro-fermentative metabolism and the fully fermentative metabolism characteristic of *rho*^*0*^ mutants, while the least sensitive were cells with fully fermentative metabolism induced by the absence of oxygen.

Yeast cells which obtain energy through aerobic respiration have extremely active mitochondria, organelles in which key pathways of aerobic respiration take place (the TCA cycle and oxidative phosphorylation associated with the respiratory chain). Although it is an efficient means of obtaining energy (about 38 ATP molecules are produced from 1 M of glucose), at the same time, due to the high ‘cost’ of production and maintenance of the activity of enzymes of these pathways and the high risk of generation of ROS as by-products of this means of respiration, these cells are more susceptible to factors disrupting their delicate redox balance^45^. The mitochondrial respiratory chain is considered one of the main sources of endogenous ROS, which explains the fact that yeast cells with aerobic metabolism have a more oxidized redox environment^46^. Thus, any factor (endogenous or exogenous) that shifts the redox balance towards oxidation reactions induces severe oxidative stress^47^. Factors which destabilize mitochondrial membranes play an important role in inducing ROS, inhibiting the activity of the electron transport chain. This means that many oxygen molecules undergo incomplete reduction, forming highly reactive oxygen radicals. Long-term production of large amounts of ROS leads to the accumulation of ROS-associated damage to DNA, proteins, and lipids, and may result in progressive cell dysfunction, which in consequence leads to the activation of the cell death programme. The results of our study confirm this scenario. The exceptional sensitivity of cells with aerobic metabolism to DMSO is most likely due to changes in the structure and function of internal mitochondrial membranes induced by this compound, which causes severe oxidative stress and also limits ATP production. Yuan et al.^42^ showed that DMSO is capable of damaging mitochondrial structures, reducing mitochondrial membrane potential, generating large amounts of ROS in the mitochondria, and inducing apoptosis in cultured astrocytes. Increased susceptibility to various types of xenobiotics in *S. cerevisiae* yeast cells with aerobic metabolism (yeast cultures in media containing a non-fermentable carbon source) compared to fermenting cells (yeast cultures in media containing surplus glucose) is not an isolated phenomenon. This pattern is also observed in the case of sorbic acid and other weak organic acids^48^, pyocyanin^49^, andrographolide^50^, selenite^51^, and imidazolium ionic liquids^52^.

There are also reports which, as in our study, indicate that *S. cerevisiae* yeast cells with fully fermentative metabolism resulting from respiratory incompetence induced by damage to mt DNA (Δ*rho*^*−*^/*rho*^*0*^ mutants) exhibit greater resistance to various xenobiotics than their parental cells with aerobic or mixed respiro-fermentative metabolism. According to Sousa et al.^53^, Δ*rho*^*0*^ mutant cells without respiratory competence displayed lower intracellular ROS accumulation and higher resistance to Pb than the wt strain. Similarly, Święciło^54^ reported that the cells of respiratory-deficient mutants (Δ*rho*^*−*^) exhibited higher resistance to sodium nitrate (V) than respiratory-proficient (*rho*^+^*)* isogenic strains.

A complete lack of oxygen forces fermentative activity in yeast and at the same time increases their resistance to the harmful effects of DMSO (Figs. 6, 7). The absence of oxygen can eliminate an important source of ROS, i.e. the active respiratory chain, which may positively influence the functions of these cells in both physiological and stress conditions. Supplementation of the medium with sterols and unsaturated fatty acids (UFAs) essential for normal growth in anaerobic conditions, which are easily taken up and utilized by yeast cells, eliminates potential growth defects in these conditions. The incorporation of these fats in the cell membranes can provide resistance to many different stress factors which *S. cerevisiae* yeast are exposed to during industrial fermentation (e.g. high osmotic pressure, prolonged hypoxic/anaerobic conditions, high ethanol concentrations and low/high temperature)^55^.

The enhanced resistance to DMSO of cells cultured in anoxia may thus be due to several phenomena. The first is direct modification of the chemical composition of yeast cell membranes following the incorporation of exogenous fats–the components of a mixture of Tween 80 and ergosterol. The second is the ability to activate a cross stress response in these conditions. The stimulus that triggers this program may be a sudden change in the means of energy acquisition from respiro-fermentation to full fermentation, the appearance of ethanol, a product of fermentative metabolism, high temperature, or ROS generated secondarily as an effect of ethanol or osmotic stress^56–58^. In all of these cases the authors noted activation of transcription factors Msn2/4p, key regulators of the general stress response, a programme which involves multiple genes and their effectors implicated in protection against damage to cell structures, repair of damaged biomolecules, import of primary and alternative energy and carbon sources, and the utilization of their intracellular reservoirs.

The data illustrated in Fig. 7 indicate that the level of toxicity of DMSO depends on the content (pressure) of oxygen in the growth environment. The lower the concentration of molecular oxygen in the growth environment, the less sensitive *S. cerevisiae* yeast cells are to the toxic effects of DMSO. In the presence of both 21% oxygen and 5% oxygen, yeast cells growing on a medium with high glucose content (2%) obtain energy by respiro-fermentation. However, the contribution of individual pathways generating ATP (glycolysis, the Krebs cycle and the respiratory chain coupled with oxidative phosphorylation) varies depending on oxygenation conditions, which is reflected in their different metabolic status^59^. Literature data indicate that as the oxygen concentration in liquid yeast cultures decreases, the amount of ATP produced in oxidative phosphorylation (respiration) relative to the amount produced by substrate phosphorylation (fermentation) successively decreases^60^. In glucose-limited chemostat cultivation with 20.9%, 2.8%, 1.0%, 0.5% and 0% oxygen in the feed gas, the amount of ATP obtained from respiration was 59%, 50%, 25% and 0%, respectively^61^. Hence it is possible that the decrease in the toxicity of DMSO which we observed as the oxygen concentration decreased was associated more with decreased respiratory chain activity (and thus a lower rate of ROS production) than with the direct interaction of oxygen with cell structure components.

The greater DMSO sensitivity of cells with impaired antioxidant defences (Δ*sod* mutants) and with an active respiratory chain suggests that the mechanisms of toxicity of this compound are mediated by ROS. To verify this hypothesis, complementation assays were performed using ascorbate, a non-enzymatic low-molecular-weight antioxidant (Supplementary Fig. S1). *S. cerevisiae* yeast growing on a standard complete medium (YPD) do not synthesize this compound, although in the presence of specific precursors they are capable of biosynthesis of ascorbic acid and its five-carbon analogue erythroascorbic acid^62^. Exogenous ascorbic acid is easily taken up by yeast cells and effectively enhances their antioxidant system^63^. In our study, however, supplementation of the medium with ascorbate at relatively high concentrations (10 and 30 mM) did not counteract the toxicity of DMSO. This is somewhat surprising, as the literature contains numerous examples of the ability of ascorbate to eliminate or mitigate the harmful effects of many types of xenobiotics with pro-oxidant properties^64–66^.

The lack of positive effects of ascorbate in the case of cells impaired by DMSO can be due to the fact that as a hydrophilic (water-soluble) antioxidant, ascorbate protects the hydrophilic environment of the cell, i.e. mainly the cytoplasmic space^67,68^, but is less effective in the hydrophobic interior of membranes, which are the main cellular target of DMSO. We noted a similar lack of protective effect of ascorbate in our earlier research on the cytotoxicity of pyrethroids (hydrophobic pesticides)^69^. Following exposure of yeast cells to the pesticides at concentrations reducing the survival rate of yeast cells, we did not observe a protective effect of this antioxidant.

## Conclusions

Our study has shown that short-term exposure to DMSO, even in a wide range of concentrations (2–20%), does not impair the functionality of cytoplasmic membranes of yeast cells of the strains tested or reduce their metabolic activity (except for the Δ*sod1* mutant and the highest concentration of DMSO). In low and intermediate concentrations, it is able to activate via Msn2p/Msn4p factors the environmental stress response (ESR), which enhances cellular protective mechanisms and in this way can modify the sensitivity of yeast to various types of external stimuli. But DMSO at relatively high concentrations may induce oxidative stress (especially in ∆*sod* mutant cells), which may be the basis for the observed toxicity of this solvent. It can significantly affect the viability of yeast cells, most likely due to the ability of DMSO to specifically inhibit the cell cycle. Long-term exposure to DMSO enhances this effect, leading to inhibition of the growth of these cells in a manner dependent on the concentration of DMSO and on the energy metabolism, antioxidant status, and genetic background of the cells. In general, cells with aerobic respiration are more sensitive to DMSO than those obtaining energy via a mixed respiro-fermentative or fully fermentative metabolic pathway. Decreased respiratory chain activity (as the oxygen concentration in the environment decreases) increases the level of resistance of cells of all strains tested to DMSO. Yeast cells with impaired antioxidant defences (Δ*sod* mutants) are more sensitive to the toxic effects of DMSO than their isogenic wild-type counterparts. This characteristic sensitivity pattern suggests that the mechanisms of toxicity of this compound are mediated by ROS. The failure of ascorbate to mitigate or eliminate the toxic effects of DMSO supports the thesis, based on our analyses, that the main site of ROS generation is organelle (mitochondrial) membranes, to which ascorbate, as a hydrophilic antioxidant, does not have access. Our results raise the question of the efficiency and safety of the use of DMSO as a solvent or a stand-alone drug in various types of cells following in vitro and in vivo administration.

## Materials and methods

Dimethyl sulfoxide (DMSO; D8418, Purity: ≥ 99.9%), resazurin (7-hydroxy-10-oxidophenoxazin-10-ium-3-one), ergosterol (3β-Hydroxy-5,7,22-ergostatriene, 5,7,22-Ergostatrien-3β-ol), Tween 80 (polyethylene glycol sorbitan monooleate), ethidium bromide (3,8-Diamino-5-ethyl-6-phenylphenanthridinium bromide), nitro blue tetrazolium (NBT) and phenylmethylsulfonyl fluoride (PMSF) were purchased from Sigma-Aldrich (Poznan, Poland). L-ascorbic acid was purchased from the Institute of Industrial Organic Chemistry (Warsaw, Poland). All other reagents, if not stated otherwise, were purchased from POCh (Gliwice, Poland) and were of analytical grade. Components of culture media were obtained from BD Difco (Becton Dickinson and Company, Spark), except for glucose and glycerol, which were obtained from POCh, (Gliwice, Poland).

### Yeast strains

The following yeast strains were used:

- SP4, a wild-type strain with the genotype MATα, leu1, arg4^70^, referred to here as wt1
- DSCD1-1C, a mutant derived from the parent strain SP4 with the genotype *MATα, leu1, arg4, sod1::natMX*^25^, referred to as Δ*sod1*
- EG103, a wild-type strain with the genotype *MATα leu2-3,112 his3∆l trp1-289α ura3-52*, referred to here as wt2, and its derivative strains containing coding region insertions TRP1 in EG110 (*sod2*∆::TRP1), referred to as ∆*sod2*.
- BY4741, a wild-type strain with the genotype *Mat a, his3Δ1, leu2Δ0, met15Δ0, ura3Δ0* Euroscarf (Frankfurt, Germany), referred to here as wt3 and its derivative strains
- ∆*msn2*, with the genotype *MAT*a**,**
- *his3Δ1, leu2Δ0, met15Δ0, uraΔ0, YMR037C::KanMX4*
- ∆*msn4*, with the genotype *MAT*a, *his3Δ1, leu2Δ0, met15Δ0, ura3Δ0, YKL062W::KanMX4*
- and double mutant ∆*msn2msn4* with the genotype *MAT*a, *his3Δ1, leu2Δ0, met15Δ0, ura3Δ0, YKR101W::KanMX4, YKL062W::loxP-NatMX-loxP*^71^.

*Rho*^*0*^ strains were generated from wild-type strains SP4 (wt1) and EG103 (wt2) and mutants DSCD1-1C (Δ*sod1*) and EG110 (Δ*sod2*) using ethidium bromide (EtB) according to the method described by Sherman et al.^72^. EtB was added to young yeast cultures grown on YPD rich medium (1% Difco yeast extract, 1% Difco Bacto peptone and 2% glucose) to attain a final concentration of 10 µg/mL. Yeast were cultured in the presence of this mutagen for 24 h with shaking at 28 °C. Then they were transplanted twice into fresh YPD medium containing EtB at the same concentration and grown in the same conditions as described above. Then the yeast were diluted in sterile water and plated onto solid YPD media to obtain single colonies. The conversion of respiratory-competent (*rho*^+^) cells to mutants incapable of aerobic respiration was verified by plating cells treated with EtB onto YPG medium containing glycerol as the only source of carbon and energy (Supplementary Fig. S2). *Rho*^*0*^ mutants are not able to grow on this medium, because glycerol is a non-fermentable carbon source for these yeasts.

### Media and growth conditions for yeasts

The yeasts were grown in liquid YPD medium containing 1% Difco yeast extract, 1% Difco Bacto peptone and 2% glucose. The cultures were grown in aerobic conditions at 22 °C for 24 h to obtain logarithmic-phase cells (LOG cells) at a density of 1–5 × 10^7^ cells/mL. Solid YPD were obtained by adding 2% agar (Difco) to liquid YPD medium and used to determine the survival of yeast cells. The YPD medium used for anaerobic cultures was supplemented with 0.5% Tween 80 as an unsaturated fatty acid (UFA) source and 0.0025% ergosterol. Solid YPGly medium consisting of 1% Difco yeast extract, 1% Difco Bacto peptone, 3% (v/v) glycerol, and 2% agar was used to verify the conversion of respiratory-competent *rho*^+^ strains into respiratory-deficient mutants Δ*rho*^*0*^ and for the culture of *rho*^+^ yeast cells, used to test the effect of DMSO on cells with aerobic metabolism. Culture conditions (applied in these studies) that allowed obtaining cells with different energy metabolism shows Supplementary Table S2.

### Short-term exposure of yeast to DMSO

LOG cells were incubated in liquid YPD medium at 22 °C for one hour with various concentrations of DMSO (0, 2, 4, 6, 8, 10, 12, 14, 16, 18 and 20% v/v), after which the yeast were centrifuged at 2,000 × g for 5 min at 25 °C. The supernatant was discarded, and the sediment containing yeast cells was re-suspended in PBS (0.1 M, pH = 7.4) buffer in such quantity as to obtain approximately 1–5 × 10^7^ cell/mL, which were examined for metabolic activity (using the resazurin reduction assay), survival rate, and catalase T activity.

The resazurin test included positive controls consisting of yeast cultures subjected to severe heat stress (induced by sharply increasing the temperature from 22 °C to 45 °C for half an hour) and oxidative stress (induced by the addition of H_2_O_2_ to a final concentration of 0.75% from a stock solution (30% v/v H_2_O_2_) for one hour). To test the ability of DMSO to induce synthesis of catalase T, positive controls were used in the form of mild heat stress (induced by sharply increasing the temperature from 22 °C to 37 °C for half an hour) and mild osmotic stress (induced by adding a 0.3 M NaCl solution for one hour).

### Determination of the metabolic activity of yeast cells using a resazurin assay

Resazurin (7-hydroxy-3H-phenoxazin-3-one 10-oxide), commercially known as Alamar Blue) is a dye commonly used to determine the numbers and viability of cells in various proliferation and toxicity assays^73,74^. Resazurin is a blue dye that is internalized by cells and metabolically reduced by diaphorase activity enzymes to the highly fluorescent pink compound resorufin. Since the oxidized form resazurin has a maximum absorption at λ = 600 nm, and the reduced form resorufin at λ = 570 nm, the ability of cells to reduce this dye can be expressed as the resazurin reduction factor (RRF). This parameter reflects the total metabolic activity, because the reducing environment can only be maintained by an efficient intermediary metabolism.

Yeast suspensions in PBS buffer obtained after centrifuging the cultures, which had previously been exposed to DMSO or subjected to strong heat and oxidative stress, were transferred in a volume of 180 µL to microplate wells, to which 20 µL of resazurin solution in PBS buffer was added (60 µM). After incubation for 1 h in darkness, the absorbance values of the solutions were read at λ = 570 and 600 nm. The RRF value was calculated as the ratio of the absorbance read at λ = 570 to the absorbance value for λ = 700 nm.

### Determinantion of catalase T activity

Cytoplasmic catalase T, like many other cytoprotective proteins in *S. cerevisiae* yeast, is an enzyme induced in response to various stress factors. The promoter of the Ctt1 gene encoding cytoplasmic catalase T contains the characteristic STRE sequence regulated by transcription factor Mns2p and its close homologue Msn4p^75^. Activity of catalase T in yeast is thus an indicator of their physiological state, intensity of stress, and thus provides a good proxy for ESR induction.

Catalase activity was estimated spectrophotometrically^76^. Briefly, 40–100 μL of yeast extract was added to 1.0 mL of 20 mM H_2_O_2_ in 50 mM potassium phosphate buffer (pH 7.0), and H_2_O_2_ decomposition was monitored at 240 nm (ε240 = 43.6 M^−1^ cm^−1^). One unit of catalase activity catalyses the degradation of 1 µmol of H_2_O_2_ per min at 25 °C. Specific catalase activity was expressed as units/mg of protein. Protein concentrations in the yeast extracts were assayed according to Bradford^77^. Extracts were prepared by disrupting yeast cells according to the procedure described by Martins & English, with minor modifications^78^. Following incubation with DMSO, yeast cells were harvested by centrifugation at 2000 × g for 5 min at 25 °C. The cells were washed twice with 100 mM potassium phosphate buffer (pH 7.0) containing 0.1 mM PMSF. The pellets were suspended in the same buffer and mixed with an equal volume of acid-washed glass beads (diameter 400–600 µm), cooled in ice for 10 min, and disrupted in a Bosch homogenizer for 2 min. Unbroken cells and cell debris were removed by centrifugation at 13,000 × g for 10 min at 4 °C. The total protein concentration in the supernatants was determined by the Bradford assay with bovine serum albumin as a standard.

### Determination of the production of superoxide radicals

Superoxide anion radical (O^∙−2^) production in yeast cells exposed to DMSO was assayed by the nitro blue tetrazolium (NBT) test^79^. NBT is reduced by superoxide anion radicals to formazan, which can be measured spectrophotometrically. To this end, 80 µL of yeast extract diluted 100 times and 920 µL 50 mM of phosphate buffer (pH 7.4) were added to 100 µL of 0.5 M NBT. At the same time, 10 µL of superoxide dismutase with activity ≥ 3.000 U/mg protein was added to an identical sample (while the volume of phosphate buffer was reduced by 10 µL). The samples were incubated for 1 h at 37 °C with shaking, after which their absorbance was measured at λ = 490 nm. The difference in absorbance for the samples with and without Sod1p is a measure of the amount of superoxide anion radical. The results were expressed as the increase in absorbance within 1 min [ΔA/min].

### Determination of yeast viability

The viability of the yeast cells was determined by plate tests, in which the yeast suspension was plated onto solid media and the number of cells capable of proliferation and colony formation was determined. Briefly, yeast cultures treated with DMSO as described above were diluted by serial decimal dilutions, and cell suspensions containing about 1–5 × 10^3^ cells/mL were plated on solid YPD medium. The inoculated plates were incubated at 28 °C for 4 days. Then the colonies were counted and their number was given as a percentage of the number in the untreated control.

### Determination of the electrical conductivity (EC) of the yeast environment

The EC of the environment in which yeast are suspended may be a marker of the functional state of cell membranes (their integrity and permeability), because an increase in its value is correlated with the increase in the concentration of ions in the environment^80^. Ions from the interior of yeast cells can be released into the environment due to cell membrane damage. Briefly, LOG cells were centrifuged at 2,000 × g for 5 min, suspended in deionized water at a temperature of 25 °C, and then treated for one hour with DMSO at concentrations of 0, 2, 4, 6, 8, 10, 12, 14, 16, 18 and 20% v/v. The use of deionized water instead of medium was necessitated by the presence of ions in the medium, which could have masked the leakage of ions from yeast cells through the damaged cell membranes^81^. Untreated yeast cells were used as a control. The positive control were cells subjected to severe heat stress according to the method described above. The EC of the medium was determined using a microcomputer conductometer CC-317 (Elmetron, Poland) and expressed as µS/cm.

### Long-term exposure of yeast to DMSO

The effect of long-term exposure to DMSO was tested by plating suspensions of LOG cells in the form of drops (spot test) on solid YPD or YPGly media to which DMSO had been added at concentrations of 0, 2, 4, 6, 8 and 10% v/v. Briefly, LOG cell cultures of yeast were diluted with PBS buffer to obtain suspensions with densities of 1–5 × 10^7^, 1–5 × 10^6^, 1–5 × 10^5^, 1–5 × 10^4^ and 1–5 × 10^3^ cells/mL, which were plated on solid YPD or YPGly medium containing 2% agar with various concentrations of DMSO using a stainless steel replicator.

In addition, complementation assays were performed to test whether the application of exogenous antioxidant agents (L-ascorbate, a reduced oxygen concentration or elimination of oxygen from the living environment) would improve yeast growth impaired by DMSO. Ascorbate is often used in this type of tests as an agent that eliminates the pro-oxidant effect of the analyzed substances^29,66,82^. In this case, in addition to DMSO, ascorbate was added to YPD media at two concentrations: 10 and 30 mM. This antioxidant was added to the agar medium cooled down to about 50 °C, just before the plates were poured. Antioxidant concentrations and methods of their application have been described in previous studies^83^. The spot-inoculated solid media were incubated in various oxygen conditions (21%, 5% and 0% oxygen) for four days in a thermostat at 28 °C. Plates with no additives (antioxidant or DMSO) incubated in standard oxygen conditions (air–21% oxygen) were used as a control. The Whitley Jar Gassing System (Don Whitley Scientific Limited, England) connected to a cylinder of anaerobic mixed gas (10 Mol% CO_2_, 5 Mol% H_2_, 85 Mol% N_2_) (Messer Polska sp. z o.o., Chorzów, Poland) was used for yeast cultivation in a controlled oxygen atmosphere. Using this device, an atmosphere containing 5% O_2_ (conditions called hypoxia) and completely oxygen-free (0% O_2_ -anoxia) was obtained. YPD medium for anaerobic cultures was additionally enriched with Tween 80 and ergosterol as described above. The spot test photos presented in Figs. 6, 7 were taken using an aCOLyte colony counter (Symbiosis, USA); the photos in Supplementary Figure S1 were taken with a Samsung smartphone, after illuminating them from below with an automatic LKB 2002 counter (Pol-Eko Aparatura).

### Statistical analysis

The results were statistically analysed using Statistica ver. 13.3 software (StatSoft, Kraków, Poland). A multivariate analysis of variance (ANOVA) was carried out at the α = 0.05 level of significance to determine significant differences between the biochemical and physiological parameters between untreated and DMSO-treated samples.

## Supplementary Information

Supplementary Information.

## Data Availability

The datasets used and /or analysed during the current study available from the corresponding author on reasonable request.
