# Supplementary material for: The effect of DMSO on Saccharomyces cerevisiae yeast with different energy metabolism and antioxidant status
Source: Sci Rep. 2024 Sep 20;14:21974. doi: 10.1038/s41598-024-72400-4 (PMC11415381; doi:10.1038/s41598-024-72400-4)
Supplement: Supplementary file 1 — Supplementary Information. [file 41598_2024_72400_MOESM1_ESM.docx]

Supplementary Information for “The effect of DMSO on *Saccharomyces cerevisiae* yeast with different energy metabolism and antioxidant status”

Agata Święciło ^1,^*, Ewa Januś ^2^, Anna Krzepiłko ^3^ and Monika Skowrońska ^4^

^1^Department of Environmental Microbiology, University of Life Sciences in Lublin, Leszczyńskiego 7, 20-069 Lublin, Poland

^2^Department of Cattle Breeding and Genetic Resources Conservation, University of Life Sciences in Lublin, Akademicka 13, 20-950 Lublin, Poland

^3^Department of Biotechnology, Microbiology and Human Nutrition, University of Life Sciences in Lublin, Skromna 8, 20-704 Lublin

^4^Department of Agricultural and Environmental Chemistry, University of Life Sciences in Lublin, Akademicka 15, 20-950 Lublin, Poland

*****Correspondence: A.Ś, [agata.swiecilo@up.lublin.pl](mailto:agata.swiecilo@up.lublin.pl)

**Supplementary Table S1.** *S. cerevisiae* strains used in this study and their characteristic.

| Name & Number | Strain symbol | Characteristic | Some phenotypic characteristics | References |
| --- | --- | --- | --- | --- |
| respiratory-competent *rho^+^* strains | | | | |
| SP4 | wt1 | a wild-type strain |  |  |
| DSCD1-1C | ∆*sod1* | a mutant lacking cytoplasmic superoxide dismutase (Cu,ZnSOD) activity | - on solid media grow more slowly than cells of wild-type strains - highly sensitive to pro-oxidant substances and high temperatures - highly sensitive to freeze-thaw stress - exhibit lysine and methionine auxotrophy, elevated free iron levels, and inactivation of enzymes containing of 4Fe-4S groups | [1]  [2]  [3]  [4] |
| EG103 | wt2 | a wild-type strain |  |  |
| EG110 | ∆*sod2* | a mutant lacking mitochondrial superoxide dismutase (MnSOD) activity | - usually less sensitive to pro-oxidant factors than Δ*sod1* mutant cells - grow slowly on media containing an unfermented carbon source (e.g. glycerol) - are highly sensitive to ethanol, high concentrations of metalloids (e.g. arsenite), salts, and high temperature | [1,3,5]  [1,2]  [1,2] |
| By4741 | wt3 | a wild-type strain |  |  |
| ∆msn2 | ∆*msn2* | a mutant lacking transcription factors (Msn2p ) which activate ESR programme | - hypersensitive to various types of environmental stress (i.e. heat stress, oxidative stress, osmotic stress, carbon source starvation) | [6,7,8] |
| ∆msn4 | ∆*msn4* | a mutant lacking transcription factors (Msn4p) which activate ESR programme |  |  |
| ∆msn2msn4 | ∆*msn2msn4* | a mutant lacking both (Msn2p, Msn4p) transcription factors |  |  |
| respiratory-deficient *rho^0^* mutants | | | | |
| Sp4 *rho^0^* | wt1 *rho^0^* | mutants obtained in this work by ethidium bromide mutagenesis, characterized by a complete lack of the mitochondrial genome | - stable during vegetative reproduction and characterized by a reduced colony size on solid media in which a fermentable carbon source is the limiting factor - permanently incapable of aerobic respiration - lack of the mitochondrial genome is not lethal provided that there is a fermentable sugar in the medium - rearrangement of energy metabolism can affect the overall sensitivity of cells to environmental factors | [9]  [10]  [10]  [10] |
| DSCD1-1C*rho^0^* | ∆*sod1 rho^0^* |  |  |  |
| EG103 *rho^0^* | wt2 *rho^0^* |  |  |  |
| EG110 *rho^0^* | ∆*sod2 rho^0^* |  |  |  |


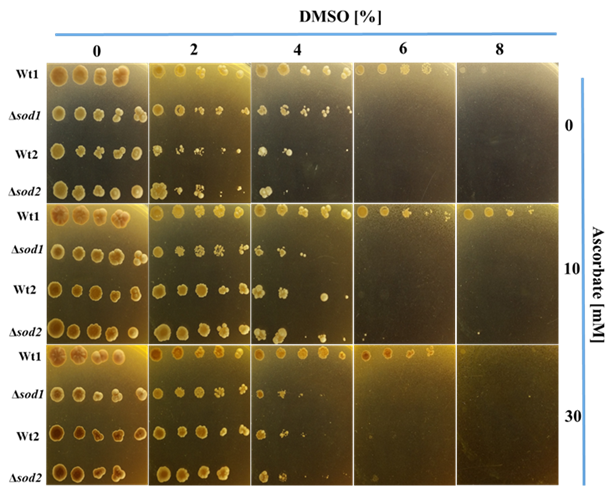


**Supplementary Figure S1.** Sensitivity of *S. cerevisiae* yeast cells with varying antioxidant status to DMSO at ascorbate presence.

Explanations: wt1, wt2, Δ*sod1*, Δ*sod2*, as in Figure 1. Representative results from a set of three experiments are shown. Ten-fold dilutions: from left to right (10^0^–10^-4^).

**Supplementary Table S2.** Culture conditions (applied in our studies) that allowed obtaining cells with different energy metabolism.

| Type of energy metabolism | Strains | Medium (carbon source), oxygen availability | References |
| --- | --- | --- | --- |
| aerobic respiration | only *rho^+^* strains | YPGly (glycerol -2%), oxygen available (21 %) | [11] |
| fermentation | *rho^+^* | YPG (glucose -2%), lack of oxygen, Tween 80 and ergosterol supplementation | [12,13] |
|  | *rho^o^* | YPG (glucose - 2%), oxygen available (21%) | [9] |
| respiro-fermentation | only *rho^+^* strains | YPG (glucose - 2%), oxygen available (5%, 21%) | [14] |


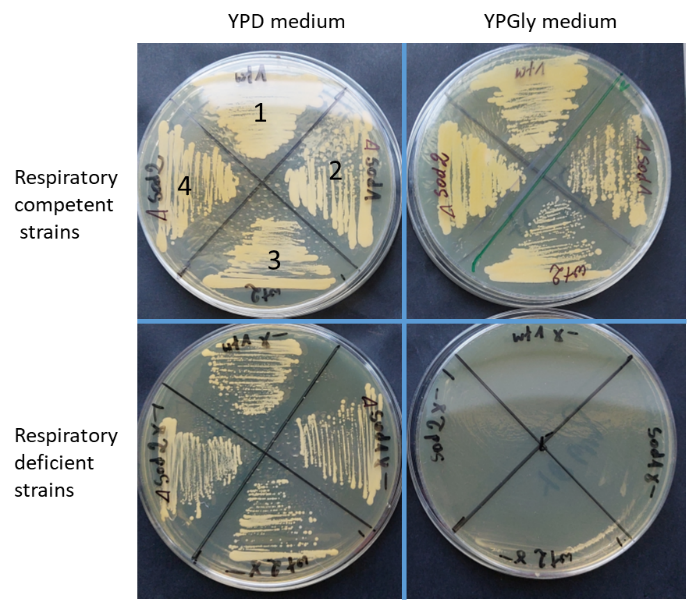


**Supplementary Figure S2.** Growth of respiratory-competent and respiratory-deficient strains on YPD and YPGly medium.

Explanations: 1- wt1, 2- Δ*sod1*, 3-wt2, 4- Δ*sod2*. The strains are in the same positions on all plates.

Supplementary References

1. Krzepiłko, A. *et al.* Ascorbate restores lifespan of superoxide-dismutase deficient yeast. *Free Radic. Res*. **2004**, *38*, 1019–1024. DOI: 10.1080/10715760410001717327
2. Dziadkowiec, D., Krasowska, A., Liebner, A. & Sigler, K. Protective role of mitochondrial superoxide dismutase against high osmolarity, heat and metalloid stress in *Saccharomyces cerevisiae*. *Folia Microbiol. (Praha)* **2007**, *52*, 120–126. DOI: 10.1007/BF02932150
3. Park, J. I., Grant, C. M., Davies, M. J. & Dawes, I. W. The cytoplasmic Cu,Zn superoxide dismutase of *Saccharomyces cerevisiae* is required for resistance to freeze-thaw stress: generation of free radicals during freezing and thawing. *J. Biol. Chem*. **1998**, *273*, 22921–22928. DOI: 10.1074/jbc.273.36.22921
4. Jensen, L., Sanchez, R., Srinivasan, C., Valentine, J. & Culotta, V. Mutations in *Saccharomyces cerevisiae* iron-sulfur cluster assembly genes and oxidative stress relevant to Cu,Zn superoxide dismutase. *J. Biol. Chem*. **2004**, *279*, 29938–29943. DOI: 10.1074/jbc.M402795200
5. Krasowska, A., Dziadkowiec, D., Łukaszewicz, M., Wojtowicz, K. & Sigler, K. Effect of antioxidants on *Saccharomyces cerevisiae* mutants deficient in superoxide dismutases. *Folia Microbiol*. *(Praha)* **2003**, *48*, 754–760. DOI: 10.1007/BF02931509
6. Estruch, F. & Carlson, M. Two homologous zinc finger genes identified by multicopy suppression in a SNF1 protein kinase mutant of Saccharomyces cerevisiae. *Mol. Cell. Biol.* **1993**, *13*, 3872–3881. DOI: 10.1128/mcb.13.7.3872-3881.1993
7. Martinez-Pastor, M. T., Marchler, G., Schüller, C., Marchler-Bauer, A., Ruis, H. & Estruch, F. The Saccharomyces cerevisiae zinc finger proteins Msn2p and Msn4p are required for transcriptional induction through the stress response element (STRE). *EMBO J.* **1996**, *15*, 2227–2235. DOI: 10.1002/j.1460-2075.1996.tb00576.x
8. Mühlhofer, M. *et al.* Deletion of the transcription factors Hsf1, Msn2 and Msn4 in yeast uncovers transcriptional reprogramming in response to proteotoxic stress. *FEBS Lett.* **2024**, *598*, 635-657. DOI: 10.1002/1873-3468.14821
9. Merico, A., Sulo, P., Piškur, J. & Compagno, C. Fermentative lifestyle in yeasts belonging to the *Saccharomyces* complex. *FEBS J.* **2007**, *274*, 976–989. DOI: 10.1111/j.1742-4658.2007.05645.x
10. Mounolou, J. L. & Lacroute, F. Mitochondrial DNA: an advance in eukaryotic cell biology in the1960s. *Biol. Cell* **2005**, *97*, 743–748. DOI: 10.1042/BC20040128
11. Hagman, A., Sall, T. & Piskur, J. Analysis of the yeast short-term Crabtree effect and its origin. *FEBS J.* **2014**, *281*,4805–4814. DOI: 10.1111/febs.13019
12. Luparia, V., Soubeyrand, V., Berges, T. Julien, A. & Salmon, J. M. Assimilation of grape phytosterols by *Saccharomyces cerevisiae* and their impact on enological fermentations. *Appl. Microbiol. Biotechnol*. **2004**, *65*, 25–32. DOI: 10.1007/s00253-003-1549-3
13. Duan, L. L. *et al.* Effects of adding unsaturated fatty acids on fatty acid composition of *Saccharomyces cerevisiae* and major volatile compounds in wine. *S. Afr. J. Enol. Vitic.* **2015**, *36*, 285–295. DOI: 10.21548/36-2-962
14. Westergaard, S. L., Oliveira, A. P., Bro, C., Olsson, L. & Nielsen, J. A systems biology approach to study glucose repression in the yeast *Saccharomyces cerevisiae*. *Biotechnol. Bioeng*. **2007**, *96*, 134–145. DOI: 10.1002/bit.21135
